# PET-Specific Parameters and Radiotracers in Theoretical Tumour Modelling

**DOI:** 10.1155/2015/415923

**Published:** 2015-02-19

**Authors:** Matthew Jennings, Loredana G. Marcu, Eva Bezak

**Affiliations:** ^1^School of Chemistry & Physics, University of Adelaide, Adelaide, SA 5000, Australia; ^2^Department of Medical Physics, Royal Adelaide Hospital, Adelaide, SA 5000, Australia; ^3^Faculty of Science, University of Oradea, 410087 Oradea, Romania

## Abstract

The innovation of computational techniques serves as an important step toward optimized, patient-specific management of cancer. In particular, *in silico* simulation of tumour growth and treatment response may eventually yield accurate information on disease progression, enhance the quality of cancer treatment, and explain why certain therapies are effective where others are not. *In silico* modelling is demonstrated to considerably benefit from information obtainable with PET and PET/CT. In particular, models have successfully integrated tumour glucose metabolism, cell proliferation, and cell oxygenation from multiple tracers in order to simulate tumour behaviour. With the development of novel radiotracers to image additional tumour phenomena, such as pH and gene expression, the value of PET and PET/CT data for use in tumour models will continue to grow. In this work, the use of PET and PET/CT information in *in silico* tumour models is reviewed. The various parameters that can be obtained using PET and PET/CT are detailed, as well as the radiotracers that may be used for this purpose, their utility, and limitations. The biophysical measures used to quantify PET and PET/CT data are also described. Finally, a list of *in silico* models that incorporate PET and/or PET/CT data is provided and reviewed.

## 1. Introduction

Anatomic imaging modalities, particularly X-ray computed tomography (CT) and magnetic resonance imaging (MRI), have long been the standard tools for the accurate localization of organs and lesions in radiation oncology. Today, they play a routine role in three-dimensional treatment planning. However, the effectiveness of structural imaging techniques in determining metabolic or functional tissue information is limited. Functional imaging has been demonstrated to be invaluable for the initial diagnosis and staging of cancer as well as the monitoring of therapy and the detection of cancer recurrence [1]. Metabolic changes in tissue commonly precede the structural changes that are detected via CT and MRI. Thus, imaging of metabolic changes may enable the detection of malignant disease at earlier stages of development [2]. In addition, the availability of functional information is advantageous for cases in which there is poor contrast between normal and malignant tissue when using structural imaging. For example, for the initial staging of lymphomas, the metabolic information provided by positron emission tomography (PET) enables more accurate delineation of the extent of nodal disease as compared with CT and bone scans. Similarly and perhaps most notably, PET demonstrates superior local staging capabilities for head and neck cancers over both CT and MRI. FDG-PET alone has a plethora of indications for a wide variety of malignancies [3]. Consequently, functional imaging modalities such as PET are playing an increasingly important role in the management of malignant disease.

PET scanning has progressed into widespread clinical practice since its first commercialization in the late 1970s. For oncological PET studies, the most utilized and extensively researched radiotracer is ^18^F-fluorodeoxyglucose (FDG). FDG-PET has demonstrated superior accuracy over conventional imaging modalities in multiple scenarios across both the diagnosis and the staging of cancer [3, 4]. In a variety of clinical settings, FDG-PET exhibits improved values of sensitivity, specificity, or both. Examples of clinical scenarios in which FDG-PET has demonstrated efficacy include the evaluation of mass lesions, the staging and restaging of cancer, the planning of radiotherapy treatments, the monitoring of therapy, and the detection of cancer recurrence [3]. However, the primary drawback of functional imaging is the lack of anatomic information that it provides. This necessitates its accompaniment with structural imaging for application in clinical oncology [1].

In order to take advantage of their inherent benefits, the combined use of both anatomic and physiologic imaging modalities is optimal. The accurate structure localization capabilities of CT complement the mapping of normal and abnormal tissue function performed by PET. Because of this, PET images are routinely read alongside CT images in order to both distinguish and localize metabolic irregularities. In the first instance, this has been achieved via the coregistration of separately acquired PET and CT images using fusion software, a technique which has especially proved to be effective for brain imaging. However, coregistration of images of other anatomical regions poses significant challenges [5]. Difficulties in the registration process primarily arise from varying patient positioning between the two image sets. Whilst such difficulties are minimal for brain scans, they may be significant in other anatomical regions wherein there may be substantial organ movement or deformation. In light of this, the development and subsequent commercialization of the PET/CT scanner in 2001 has generally addressed the issues affecting the coregistration of separately acquired PET and CT images [6].

The use of integrated PET/CT in oncologic imaging has since become a widespread field of research, particularly with the utilization of ^18^F-FDG. This combined modality overcomes some of the drawbacks that are characteristic of standalone PET scanning, namely, the significant presence of noise in attenuation correction factors, the lengthy duration of scans, and the absence of anatomic markers [7]. While the vast majority of published research concerns standalone PET, PET/CT has begun to show great promise across a multitude of clinical settings [3]. Studies have shown significantly improved accuracy in the staging of nonsmall cell lung cancer with PET/CT over the separate performance of PET and CT [8–10]. Some centres perform a series of PET/CT scans on nonoperative head and neck cancer patients following either radiation therapy or chemotherapy due to its indispensable combined anatomical and functional information [3, 7, 11]. For patients with recurrent or metastasized thyroid cancer, the localization capabilities of ^18^F-FDG-PET/CT can lead to improved diagnostic accuracy [12]. Finally, the incorporation of FDG-PET/CT data into the radiation treatment planning process has repeatedly shown to improve target delineation and enhance the therapeutic ratio via its increased cancer staging accuracy [13]. The ongoing clinical evaluation and further innovation of PET/CT, namely, via technological improvements and the establishment of new tracers, will continue to propel this technology into widespread use in oncology [3].

The significant interpatient variability in both tumour behaviour and normal tissue response to cancer therapy has prompted increasing demand for individualized treatment planning. Simulations of radiobiological processes in malignancies provide scope for the further optimization of individual treatment plans and to ultimately improve patient outcomes. The development of computer or* in silico* models of these radiobiological processes is particularly beneficial [14]. It is here in which PET and PET/CT, as noninvasive functional imaging modalities, can play a crucial role [15–17]. The aim of this work is to review the application and effectiveness of PET and PET/CT for the* in silico* modelling of tumours. In particular, it examines the various parameters that can be determined via PET and PET/CT imaging along with the assortment of radiotracers available for these purposes. The biophysical quantities used to quantify PET and PET/CT data, including SUV and compartmental models, are assessed. Finally, an overview of the existing* in silico* models that utilize PET or PET/CT data is provided.

## 2. Tumour Model Parameters Obtainable from PET Imaging

With the objective of optimal, individualized treatment planning, many biological characteristics of a given tumour can be considered for computer simulation. In the case of radiation therapy, the proliferative potential of a tumour and its radiosensitivity are of particular interest. Parameters which describe tumour cell proliferation and repopulation characteristics are useful for* in silico* modelling of tumour growth and treatment response. Similarly, since it is well established that tumour's oxygenation level contributes significantly to its radioresistance, modelling parameters that characterize tumour hypoxia and angiogenesis are also common. Increasingly,* in silico* models have expanded to incorporate additional contributing factors to tumour radiosensitivity, including intracellular tumour pH, gene expression, and cell-cycle simulation. The complexity of any given model dictates the number of characteristics simulated, as well as the accuracy with which each tumour characteristic is simulated. Simpler models may use averaged, macroscopic measures of a given tumour characteristic, while more complex models may simulate a tumour and its behaviour at the microscopic, molecular, and even atomic levels. Indeed, some of the most accurate, contemporary models are “multiscale”; they simulate tumour behaviour across multiple biological scales, reconciling the macroscopic and microscopic levels [18]. Details of the various parameters utilized for tumour modelling obtainable from PET and PET/CT data are provided below.

### 2.1. Cell Proliferation

There are many approaches to modelling cell proliferation but it is useful to separately consider modelling at the macroscopic and microscopic scales. At the macroscopic scale, tumours are commonly simulated using continuum models. Using this approach, gross tumour morphology and behaviour are modelled under various environmental conditions and typically governed by a set of differential equations whose initial conditions serve as input parameters [18]. Input parameters include such quantities as cell density (both viable and necrotic), cell volume fractions, and gross tumour proliferation and metabolic rates [19]. Such parameters may be gathered from PET/CT data, where a corresponding radiotracer can be utilized [20]. Details of various radiotracers and the functional information they provide are given in Section 3.

In contrast to continuum tumour models, discrete tumour models developed at the microscopic scale simulate individual cell behaviour. Their input parameters correspondingly describe a set of biophysical rules applied to the modelled cells and the diversity of these rules varies across different models. Each cell is assigned an initial state and its status is subsequently tracked throughout the simulation. Consequently, discrete models directly simulate cell proliferation; a process which is mediated by input parameters such as proliferative potential, cell-cycle positions, and durations for different cell types, probability of cell division, cell loss rates, and maximum tumour radius or cell number [19]. Patient-specific parameters such as tumour radius and proliferating cells per voxel are directly obtainable from functional imaging data [15].

### 2.2. Hypoxia

The adverse effects of tumour hypoxia on patient outcomes following radiotherapy have been well established. Indeed, hypoxia is often attributed to poor tumour control probabilities in locally advanced head and neck cancers, for which low oxygenation levels are common [21]. Accordingly the simulation of oxygenation in tumours has been a prolific area of research in the tumour modelling community for more than 60 years [22]. The simulation of tumour hypoxia varies greatly across different models; the most complex models concurrently simulate oxygen diffusion, oxygen consumption by tissue, and the interdependence of oxygen with tumour proliferation and vasculature [23].

Oxygen information is generally modelled using an analytic approach. That is, sets of differential equations are used to describe oxygen diffusion and/or consumption rates both during tumour growth and in response to treatment. Even stochastic (discrete) tumour cell proliferation models that incorporate oxygen information typically employ a composite approach; oxygen and other substrate concentrations are generally governed by continuous fields. Since the oxygen distribution within a tumour is directly related to the extent and nature of its vasculature, many tumour hypoxia models incorporate blood vessel information into their simulations. The most sophisticated models simulate angiogenesis (see Section 2.3) and its effect on tumour hypoxia [16, 23, 24].

Simulated oxygen distributions are typically quantified using partial oxygen tension or pO_2_ values. Thus, for the successful simulation of tumour hypoxia, realistic initial pO_2_ conditions must be set. In order to categorize the oxygen status of tissue, binary approaches to oxygen modelling establish a defined threshold pO_2_ value below which the region is considered to be hypoxic. More robust models establish a more detailed relationship between tumour growth or treatment response and oxygen information and may incorporate both oxygen diffusion and oxygen consumption by tissue [23, 25]. Additional parameters of interest may be included for oxygen effects, such as reoxygenation probability distributions and hypoxic thresholds for which cell quiescence is initiated. Multiple studies have demonstrated the utility of PET for obtaining pO_2_ distributions for use in patient-specific tumour models [16, 23, 26].

### 2.3. Angiogenesis

The extent and nature of tumour vasculature significantly influence tumour growth and oxygen status. Consequently, the simulation of angiogenesis serves as a useful complement to models of tumour cell proliferation and hypoxia. Likecell proliferation, models of tumour-induced angiogenesis may be either continuous or discrete in nature. More advanced models utilize both approaches for simulating the various mechanisms involved in angiogenesis, such as capillary sprout formation, endothelial cell migration, blood flow, and vessel adaptation [19].

Input parameters related to vessel branching generally include probabilities of random endothelial cell migration, chemotaxis with tumour angiogenesis factor concentration (especially vascular endothelial growth factor or VEGF), and haptotaxis with fibronectin gradients [18]. The utility of PET for the imaging of specific angiogenic markers, such as*α*_*v*_ *β*_3_ integrins and tumour expression of VEGF, has been demonstrated by multiple groups [28, 29]. To date, tumour models of angiogenesis have traditionally not incorporated angiogenesis-specific PET data. However, since cellular oxygenation and tumour vasculature are intimately related phenomena, hypoxia-specific PET data has been utilised in models of tumour vasculature [16, 23]. Specifically, the value of PET information for the conversion of oxygen maps into capillary density maps has been demonstrated. The potential of angiogenesis-specific PET-based imaging for input in modelling the temporal development of vasculature or angiogenesis is well understood and will develop with ongoing studies [16].

### 2.4. pH

Though the intracellular pH of solid tumours is maintained in a range similar to that of normal cells, the extracellular pH of solid tumours is commonly acidic. The increased glucose metabolism of solid tumours, assisted by characteristically poor perfusion, is the most probable cause of their low extracellular pH. This is because glucose catabolism results in net acid production and insufficient vasculature cannot remove excess acid from the extracellular environment [30].

Perhaps the most compelling value of including pH in tumour growth models arises from the acid-mediated tumour invasion hypothesis. This suggests that tumour cells develop phenotypic adaptations to the harmful effects of acidosis during carcinogenesis, traits that are not present in normal cells. Consequently, tumour cells are rendered relatively impervious to the decreased pH in the tumour microenvironment resulting from increased anaerobic glycolysis, which is otherwise toxic to normal tissue. Effectively the tumour provides for itself a selective growth advantage and a useful mechanism for invasion [31]. Investigation of this phenomenon is particularly suited for* in silico* tumour growth modelling and the role played by acid gradients in triggering tumour invasion has been evaluated this way [31]. Excess H^+^ ion concentration may be simulated in a continuum model utilizing reaction-diffusion partial differential equations, where input parameters such as acid production rate, reabsorption rate, and H^+^ ion diffusion coefficients may be obtained from measurement [32].

As with angiogenesis,* in silico* models incorporating tumour pH have not utilized noninvasive PET data for input. Though PET has been used for the measurement of pH since the 1970s and was the first noninvasive* in vivo* pH meter, it has historically been both an inaccurate and imprecise measurement tool [30]. However, the development of novel radiotracers that selectively target acidic tumours will enable the incorporation of pH related PET data into* in silico* models of tumour growth [33].

## 3. Radiotracers Used for Tumour Modelling

PET and PET/CT are able to image an increasing variety of physiological phenomena. This versatility arises from the ability to select a radiotracer that specifically targets a particular mechanism. Additionally, the diversity of PET tracers continues to expand with ongoing innovations in radiopharmaceutical production. Today, radiotracers exist for the imaging of metabolism, proliferation, perfusion, drug/receptor interactions, and gene expression. Despite this variety, the extent of PET data incorporated into* in silico* tumour models has so far been limited to radiotracers specific to glucose metabolism, cell proliferation, and hypoxia. There is significant potential for the use of alternative radiotracers to obtain additional functional information for* in silico* models using PET. A list of radiotracers whose information has been directly incorporated into* in silico* models as well as those that show significant promise for such applications is provided in Table 1.

### 3.1. FDG

^18^F-2-Fluoro-2-deoxy-glucose or FDG is by far the most commonly used and extensively researched PET radiotracer. Today, FDG-PET plays an important role in oncology. It has been recommended for use as an imaging tool additional to traditional radiological modalities in the appropriate clinical setting. In particular, it has demonstrated efficacy in the diagnosis, staging, unknown primary discovery, and the detection of cancer recurrence [1].

Increased glucose consumption is a typical characteristic of most cancers. In hypoxic regions, the Pasteur effect results in the upregulation of anaerobic glycolysis and the GLUT 1 glucose transporter in tumour cells. However, even if oxygen is plentiful, cancers undergo accelerated glycolysis. This observation, called the Warburg effect, is widely attributed to mutations in oncogenes and tumour suppressor genes [34]. Since FDG is a glucose analogue, it is a particularly suitable radiotracer to measure the increased glucose utilization typical of cancers. Along with increased glucose consumption, the upregulation of appropriate enzymatic activity further amplifies FDG uptake in tumour cells.

The primary drawback of FDG-PET for oncologic imaging is that FDG uptake is not specific to cancer. That is, FDG-PET exhibits a poor level of specificity for certain applications. FDG uptake may be intense in benign diseases as well as in areas of infectious disease and inflammatory tissue. That is, there are many potential causes of false-positive PET signals in oncologic imaging [1, 35, 36]. Conversely, some malignant diseases do not exhibit high glycolytic activity. Bronchioloalveolar carcinoma and carcinoid tumours are examples of cancers for which false-negative signals may occur for standalone FDG-PET imaging [35]. Combining FDG-PET with other imaging modalities has served to mediate this drawback somewhat; FDG-PET/CT has demonstrated superior performance than standalone FDG-PET in common cancers [37]. Additionally, the emergence of novel radiotracers that target biochemical processes that are more specific to cancer promises to overcome the relative nonspecificity of FDG-PET in oncologic imaging.

Commensurate with the predominance of FDG as the PET radiotracer of choice, metabolic information provided by FDG-PET is often utilized in* in silico* models of both tumour growth and treatment response [16, 20]. The specific information employed from FDG-PET varies across such models. Images may be used solely to identify existing cancerous tissue, particularly in simulations for the prediction of tumour response to therapy [16]. Alternatively, glucose metabolism data from FDG-PET can be quantitatively used to simulate metabolic processes in predictive models of tumour growth [20].

### 3.2. FLT

PET radiotracers specific to cell proliferation are an effective alternative to those specific to glucose metabolism, such as FDG. Of these tracers, ^18^F-3-fluoro-3-deoxy-thymidine (FLT) is perhaps the most researched and the most utilized. FLT-PET is typically a less sensitive imaging modality than FDG-PET: the difference in FLT uptake between normal and malignant tissues is usually less pronounced than that for FDG [38]. However, FLT uptake correlates very well with Ki-67, an index of cell proliferation. Consequently, FLT-PET is useful for aiding in the grading of tumours. The combined FLT-PET/CT modality has demonstrated efficacy for the early prediction of treatment response [39–41] as well as the assessment of cancer aggressiveness [42]. The uptake of FLT in infectious or inflammatory tissue is less than that of FDG and FLT has lower background activity in the brain and thorax [43, 44]. Consequently, the specificity of FLT-PET/CT exceeds that of FDG-PET/CT in certain imaging applications [44].

The magnitude of FLT that is trapped in a tumour cell is proportional to the amount of DNA/RNA synthesis undertaken by the cell. Since the growth of malignant tissue is intricately related to DNA replication, the degree of FLT uptake is strongly correlated with proliferation rate. Accordingly, FLT-PET data is particularly useful for patient-specific tumour modelling, where proliferative rates are often of paramount interest. A group led by Benjamin Titz at the University of Wisconsin has correspondingly acquired cell proliferation information from FLT-PET data for* in silico* models of tumour growth and treatment response [15, 16].

### 3.3. FMISO

In an effort to develop accurate, noninvasive measurement techniques of tumour hypoxia, a number of PET radiotracers have been produced which irreversibly bind to cells in poorly oxygenated conditions. Of these, ^18^F-fluoromisonidazole (FMISO) is the most extensively studied and clinically validated. FMISO uptake is inversely proportional to O_2_ level and perfusion does not restrict its delivery to malignant tissue. Several studies have demonstrated FMISO-PET to be a viable prognostic indicator of tumour response to treatment [45].

FMISO-PET has not been universally adopted into routine clinical application because of a number of limitations inherent in the radiotracer. Since it relies on passive transport mechanisms, its uptake is relatively slow in hypoxic tumours, usually requiring 2–4 hours to be selectively retained in the target following injection [46]. FMISO-PET imaging also exhibits relatively low tumour-to-background ratios, since its binding to malignant, hypoxic cells is highly nonspecific. Finally, considerable levels of unwanted, radioactive metabolite products result from the nonoxygen dependent metabolism of FMISO [45]. Improvements in the quantification of hypoxia by modelling FMISO-PET dynamics, as opposed to using the standardized uptake value (SUV) in a binary manner, may aid in overcoming contrast limitations. Several studies have demonstrated reasonable success in the simulation of tracer transport and its application to tumour models (see Section 5) [25–27].

### 3.4. Cu-ATSM

Alternative hypoxia-specific PET radiotracers have been developed in order to overcome the various limitations of FMISO. Several other nitroimidazole compounds have been developed for this purpose [47]. In 1997, an alternative hypoxia PET tracer was proposed that does not suffer from the undesirable radioactive residues of nitroimidazoles. This tracer is Cu(ll)-diacetyl-bis(N^4^-methylthiosemicarbazone) or Cu-ATSM [48]. Cu-ATSM has evolved to become one of the most promising PET agents for hypoxia imaging. It has demonstrably high hypoxic tissue selectivity [45]. It is able to rapidly identify hypoxic tissue with high tumour-to-background ratios, due to a combination of small molecular weight, high cell membrane permeability, rapid blood clearance, and prompt retention in hypoxic tissues [45].

The effectiveness of Cu-ATSM for providing clinically relevant tumour oxygenation information has been confirmed in multiple studies and its predictive value of tumour behaviour and treatment response has been demonstrated [49–51]. Perhaps unsurprisingly, it is a preferred radiotracer for the determination of oxygenation information using PET for incorporation into* in silico* tumour models [15, 16, 23]. For example, Titz and Jeraj chose a sigmoidal relationship between the SUV of Cu-ATSM and local tissue oxygenation [15], following the findings of Lewis et al. [52]. Using pretreatment Cu-ATSM-PET spatial maps of tumour oxygenation, it was demonstrated that lower oxygen levels resulted in reduced treatment efficacy. However, the group did note that further investigation into the quantitative relationship between partial oxygen tension and Cu-ATSM uptake is warranted.

### 3.5. Other Radiotracers

Although* in silico* models are yet to incorporate PET or PET/CT information beyond glucose metabolism, cell proliferation, and tumour oxygenation, there is scope for the use of tracers that image additional processes. Tumour acidosis arising from amplified glycolysis is a common feature of cancers and is a likely trigger of invasion into surrounding tissue [53]. Consequently, several mathematical models, inclusive of tumour acidity, have been developed to study the glycolytic phenotype and the tumour-host interface [31, 54]. There is suggestive potential of PET and particularly PET/CT for directly obtaining parameters of interest for such models, including glucose metabolic rate and acid production rates. One promising, novel PET tracer that specifically targets acidosis is pH low insertion peptide (pHLIP) [33]. pHLIP binds to acidic cell membranes and has demonstrated ability to target areas of hypoxia and carbonic anhydrase IX (CAIX) overexpression, an acid-extruding protein [55].

As discussed in Section 2, the simulation of angiogenesis is of significant interest for* in silico* tumour modelling. Though parameters related to angiogenesis can be indirectly obtained using hypoxia-specific PET-imaging, alternative markers of angiogenesis can instead be targeted. For example, vascular integrins are targeted by PET radiotracers containing the tripeptide sequence arginine-glycine-aspartic acid (RGD) [28]. In particular, the*α*_*v*_ *β*_3_ integrin is a receptor related to cell adhesion and involved in tumour-induced angiogenesis that can be imaged using radiotracers such as ^18^F-Galacto-RGD [29].

In models that simulate specific tumour types, PET information from alternative tracers might be useful. For example, neuroendocrine tumours are typically characterised by an increased L-DOPA decarboxylase activity [56]. The imaging of advanced neuroendocrine tumours has been validated with PET using ^18^F-dihydroxyphenylalanine (FDOPA) [57] and may be of value in the modelling of these tumours. Similar arguments may be made for the simulation of breast cancers and the imaging of oestrogen receptor expression using ^18^F labelled oestrogens such as ^18^F-fluoroestradiol (FES) [58].

## 4. Biophysical Parameters Used in PET and PET/CT

The integration of reliable imaging-based information into* in silico* models of tumour growth and treatment response greatly relies on the accurate and precise quantification of imaging data. This is especially relevant for PET and PET/CT, for which radiotracer uptake is dependent on a host of factors. SUV is the most extensively used parameter clinically for the analysis of PET tracers, but its high degree of sensitivity to multiple variables can render the comparison of SUVs taken at different times or between different centres to be extremely difficult [59].

Details of the biophysical parameters used to quantify PET and PET/CT data are provided below. Their applications and limitations are discussed and compared, as well as the various methods that have been developed to overcome the potential pitfalls of a given measure. The present use of PET and PET/CT quantification measures in* in silico* models of tumour growth and treatment response is also discussed.

### 4.1. SUV

The standardized uptake value is the quintessential parameter employed to analyse and quantify PET radiotracer data. It is defined as follows:

(1) $$\begin{array}{c} \text{SUV}=\frac{\text{radiotracer}\;\text{concentration}\;\text{in}\;\text{ROI}}{\text{total}\;\text{injected}\;\text{activity}/N}\;\;\text{g}/\text{mL}, \end{array}$$

where the concentration is as measured with PET in kBq/mL, ROI is the region or volume elements of interest, and *N* is a factor normalizing for body weight, body surface area, or lean body mass. The overall denominator has units kBq/g. The radiotracer is commonly computed by scanning the patient for a 5–15-minute interval after a predetermined period (e.g., 1 hour) after radiotracer injection.

In general, SUV depends on the time between injection and scanning as well as multiple image acquisition settings such as the reconstruction algorithm and scatter and attenuation corrections [59]. Its comparative value is hampered by methodology differences, such as choice of normalization factor and choice of* max*,* mean*,* peak*, or* total* SUV [60]. SUV may also be confounded by biological mechanisms, such as variations in plasma clearance before and after treatment and plasma glucose concentration (in the case of FDG) [59].

Despite its limitations, SUV poses advantages over alternative quantification techniques such as compartmental methods (see Section 4.2). It is the only method of PET quantitative analysis that can be realistically employed for routine clinical use, due to its sheer simplicity and the efficiency of the associated scan protocols. In addition, the effectiveness of SUV for the assessment of cancer therapy response by comparing values for scans taken before and after treatment has been extensively validated. This is particularly true for FDG-PET, whose efficacy has been confirmed for multiple cancer types [59]. Accordingly, in spite of its large variation in some situations, the use of SUV is common for the acquisition of metabolic information, proliferation rates, and pO_2_ values for use in* in silico* tumour models [16, 20, 23].

### 4.2. Compartmental Models

Compartmental or kinetic modelling (CM) is the “gold standard” of quantification methods for PET data [59]. In CM, the exchange of the PET radiotracer between a number of physiological entities (called compartments) is simulated. These compartments are homogeneous in nature and the tracer transport and binding rates between them is modelled by a set of first-order differential equations [61]. The set of equations are solved numerically to obtain the rate constants, kinetic parameters analogous to those outlines in Section 2, such as glucose metabolic rates or blood flow [59].

Despite its accuracy and relative independence of confounding effects as compared to SUV, CM has the disadvantage of requiring a complex, time-intensive acquisition protocol. Techniques with which the requisite scan protocol complexity of CM can be overcome CMare an ongoing field of research [62, 63]. In the case of radiotracers such as FDG for which the use of SUV has been strongly validated (via comparison with CM, in some cases), the added benefit of employing CM techniques for PET analysis is likely to be insubstantial [59].

However, in the case of FMISO-PET imaging of hypoxia, the use of CM is warranted. Whilst FMISO can be used to identify and image tumour hypoxia, it typically exhibits poor tumour-to-background ratios using the standard SUV measure, generating highly variable results [25, 27]. Compartmental modelling of hypoxia imaging for dynamic FMISO-PET data has shown great success in ameliorating this problem, with particularly promising contributions from Thorwarth et al. [25] and Wang et al. [27].

### 4.3. Hounsfield Units

The process of positron emission tomography is based on the coincident detection of colinear, 511 keV photons originating from an annihilation event. This measurement may be affected by an interaction between one or both of the photons and the attenuator prior to reaching the detectors. A colinear coincident event is consequently not detected and may instead register as scatter coincidence or no coincidence. To account for this signal loss, attenuation corrections are performed during PET image reconstruction in an effort to salvage the true radiotracer distribution. Until the development of PET/CT attenuation correction in PET was performed using a transmission scan taken immediately prior to the imaging scan, effectively doubling the total scan time. In modern PET/CT scanners, CT-based attenuation correction of PET images can be performed using the immediately available CT images. 511 keV linear attenuation values are obtained from the Hounsfield unit (HU) data provided by the CT using an appropriate transformation scheme [64], usually a bilinear relationship.

CT scanners convert attenuation coefficient distributions *μ*(*x*, *y*, *z*) into HU for display. Since attenuation is directly proportional to attenuator density, the HU of a particular voxel may be interpreted as the density of the object within that voxel relative to that of water. Typical scans consist of image noise within the range of 10–50 HU, corresponding to a relative error of 1–5% [65]. Consequently, CT is a powerful imager of tumour density. For oncologic scenarios in which lesions and background tissues are characterized by similar HU values, assessment with CT is facilitated by the use of positive and negative contrast agents. Contrast enhancement in PET/CT has been reported to improve lesion detection, characterization, and localization in some clinical settings [66–68].

Positive contrast agents within PET/CT may cause overestimation of PET attenuation with contrast-enhanced CT based attenuation corrections. This can lead to artifacts of apparently increased tracer uptake in regions of high contrast concentration within the PET image. However, such artifacts can often be attributed to an underlying vessel and hence do not cause problems with image interpretation [65]. Furthermore, several research studies have confirmed the clinical insignificance of this effect, since the typical SUV measure is negligibly affected by contrast [69, 70].

## 5. Review of Computational and Mathematical Models of Tumour Growth and Prediction to Treatment Response Based on PET Imaging Data

With the advances in technology, the current imaging modalities offer a great variety of biological, biophysical, and clinical parameters to be further studied and implemented into complex tumour models. Computational modelling is an ever-increasing area of research in tumour biology and therapy. Depending on their design (i.e., continuum or discrete) models offer various levels of understanding of biological, biochemical, and biophysical processes occurring in tumours before and during treatment. Models are versatile in terms of input parameters, equations used, phenomena simulated, and end points. While never perfectly illustrating the biological reality, models are valuable complements to kinetic analysis of tumour growth and development, treatment outcome prediction, patient selection, and important decision-making towards personalized medicine.

There are a large number of computational and mathematical tumour models that incorporate functional imaging data in the scientific literature. This is particularly true for PET and PET/CT. A detailed list is provided in Table 2, which includes models of tumour growth, tumour characteristics, and response to treatment. The aims of the models, the corresponding imaging techniques used and physiological parameters imaged, and the relevant group's findings are given.

Tumour growth models are an important initial step when modelling treatment response. Using dual-phase CT and FDG-PET imaging modalities, Liu et al. have developed a tumour growth model for pancreatic cancers [20, 71]. They have introduced the intracellular volume fraction (ICVF) as biomarker for the estimation and evaluation of the model's parameters, based on longitudinal dual-phase CT images measured on pre- and postcontrast images. SUV was used as a semiquantitative measure of tumour metabolism (metabolic rate), which was further related to tumour proliferation rate. The model was validated by comparing the virtual tumour with a real pancreatic tumour, in terms of average ICVF difference of tumour surface, relative tumour volume difference, and average surface distance between the predicted tumour surface and the CT-segmented (reference) tumour surface [20].

Perhaps the vast majority of the models address the challenge of tumour hypoxia and neovascularization [15, 16, 23, 27]. The approach used in the models varies among research groups. Given that compartmental models are great tools in kinetic modelling of perfusion, diffusion, and pharmacokinetics of various tracers, they were chosen by some groups to quantitatively estimate the levels of hypoxia in head and neck tumours and also to assess the hypoxic distribution within the tumour [25, 27]. Considering the controversies around SUV and its correlation with the partial oxygen tension (pO_2_), Thorwarth et al. came to a practical conclusion, whereby compartmental kinetic models are more reliable for hypoxia assessment than early static SUV measurements, due to the low uptake of FMISO by severely hypoxic cells.

Experimental probability density functions were employed by other groups to simulate the direction and spatial arrangement of microvascular tumour density, using patient-specific PET imaging information [23]. The discovered correlation between microvessel density and tumour oxygenation levels (i.e., pO_2_) suggests that patient-based simulation can contribute towards individualized patient planning and treatment.

Hybrid models (or multiscale models) are often used for complex assessment of tumour growth and behaviour under therapy, due to their versatility and ability to integrate mathematical/computational modelling with experimental data on different physical scales. The hybrid model developed by Titz and Jeraj is an example of this [15]. Depending on the input parameters chosen in terms of relevance and reliability, such hybrid models can predict, with high accuracy, tumour response to various treatments. As illustrated in Section 3, functional imaging and particularly PET imaging employing tumour-specific radiotracers play an important role in fulfilling this task. Therefore, information regarding tumour kinetics and proliferation can be obtained from proliferation-specific agents (such as FLT) while oxygen distribution data is gained from hypoxia-specific radiotracers (such as FMISO or Cu-ATSM). Additionally, with ongoing radiotracer development and evaluation, there is scope for obtaining additional tumour characteristics, such as acidosis using pHLIP and gene expression using protein-specific agents such as Galacto-RGD, FDOPA, and FES.

To further prove the usefulness of complex multiscale models, the same group has simulated the effect of bevacizumab, an anti-VEGF agent, which is administered for targeting endothelial cell population in tumours [16]. Tumour hypoxia and proliferation data were gathered from PET images taken before and after the antiangiogenic treatment. Simulated hypoxia levels were compared with mean SUV values and changes in mean SUV after the administration of bevacizumab for various levels of hypoxia, proliferation, and VEGF expression were analysed. The findings were implemented on imaging data of a phase I clinical trial that involved eight head and neck cancer patients, showing the potential of such models to optimise treatment outcome.

## 6. Conclusion

The incorporation of patient-specific data into multiscale models is necessary for individualized, predictive simulation. This is an essential component of predictive oncology. Image-based information can be transformed into input parameters and incorporated into either probabilistic or deterministic equations governing their relationships and interdependences. Using these tools, countless hypotheses can then be generated and scenarios of “what if” can be simulated and solved. Models usually have the benefit of independence from the manner in which input parameters are obtained. This allows for the constant refinement of parameters with future innovations in measurement techniques, particularly in PET and PET/CT. Additionally, most models can be readily adapted to include new parameters in order to better resemble the real tumour environment. The widespread, continuing research into* in silico* model development and refinement permits the simulation of cancer with ever-increasing accuracy, with the goal of optimally individualizing cancer management and improving overall patient outcome.

## Figures and Tables

**Table 1:** PET Radiotracers whose data has been/the potential to be incorporated into *in silico* models.

PET radiotracer	Functional characteristic	Corresponding tumour model parameters	Use in *in silico* models?
FDG	Glucose metabolism	(i) Intracellular Volume Fraction (ICVF)	Yes
		(ii) Acid production rates^*^	

FLT	DNA replication	Tumour cell proliferative rates (vector- or voxel-based)	Yes

FMISO	Hypoxia	(i) Partial oxygen tension (pO_2_)	Yes
		(ii) Relative hypoxic fraction (RH)	

Cu-ATSM	Hypoxia	Partial oxygen tension (pO_2_)	Yes

pHLIP	Acidosis	Extracellular pH (pH_e_)	Potential

Galacto-RGD	Angiogenesis	(i) *α*_*v*_ *β*_3_ expression rate	Potential
FDOPA	Malignancy	(ii) L-DOPA activity	Potential
FES	Malignancy	(iii) Oestrogen overexpression	Potential

^*^The specificity of FDG to glucose metabolism provides in indirect measure of acid production rates in tumour cells, since anaerobic glycolysis is net acid producing.

**Table 2:** Models of tumour growth and prediction to treatment response based on PET imaging data.

Aim of the model [references]	Imaging technique used	Model parameters	Results/observations
Models of tumour growth			
Spatial-temporal characterization of pancreatic tumour growth and progression [20]	Dual-phase CT and FDG-PET	Intracellular Volume Fraction (ICVF) which reflects tumour cell invasion and SUV used for determination of cell metabolic rate, growth rate, cell motion: diffusion and advection (for mass effect).	The model was successfully validated against a real tumour using average ICVF difference of tumour surface, relative tumour volume difference & average surface distance between predicted and segmented tumour surface.

Models of tumour characteristics			
Evaluation of tumour hypoxia in head and neck tumours [25]	Dynamic FMISO-PET	Tracer transport and diffusion model; voxel-based data analysis used to decompose time-activity curves into components for perfusion, diffusion and hypoxia-induced retention.	Quantification of hypoxia; hypoxic regions are spatially separated from blood vessels; tracer uptake occurs in viable hypoxic cells-only. The kinetic model is more accurate than static SUV values.
Simulation of tumour oxygenation [26]	Dynamic FMISO-PET	Model input parameters for steady-state O_2_ distribution: 2D vascular map, oxygen tension and rate of oxygen consumption. Binding rates of FMISO estimated and spatial-temporal O_2_ distribution found. Probability density function was used to model tumour vasculature to identify hypoxic sub-regions.	Hypoxic sub-region distribution and shape resulting from the simulation agree with real imaging data. It was shown that the extent of vasculature is of greater importance than the level of tissue oxygen supply. The model allows for quantitative analysis of tumour parameters when physiological changes occur in tumour microenvironment.
Estimation of tumour hypoxia in head and neck tumours [27]	Dynamic FMISO-PET	Region of interest and arterial blood are identified via PET. Values of kinetic parameters (for oxic, hypoxic and necrotic areas) are taken from PET-scanned patient data.	Voxel-based compartmental analysis is feasible to quantify tumour hypoxia and more reliable than static PET-SUV measurements.
Simulation of tumour vasculature [23]	Cu-ATSM PET and contrast CT	Capillaries were simulated using probability density functions (micro-vessel density) and patient imaging data. Capillary diameter was modelled in conjunction with voxel size; a relationship between vessel density and pO_2_ was employed.	Simulation of homogenous and heterogeneous oxygen and vascular distribution. The model was tested on mouse tumour: the simulated vasculature and the Cu-ATSM PET hypoxia map represent the image-based hypoxia distribution. The model can be used for anti-angiogenic treatment simulation.

Models of treatment response			
Tumour growth and response model with hypoxia effects [15]	^ 18^F-FLT (for proliferation) & Cu-ATSM PET (for hypoxia) and CT	CT used for tumour anatomy. Behaviour of tumour voxels modelled upon PET data. FLT uptake was used as proliferation index. A sigmoid relationship was considered between Cu-ATSM SUV and pO_2_. The Linear Quadratic model was used for cell survival.	The model accurately reproduced tumour behaviour for different oxygen distribution patterns. Treatment simulations resulted in poor control for hypoxic tumours: heterogeneous oxygen distribution resulted in heterogeneous tumour response (i.e. higher survival among hypoxic cells).
Evaluation of tumour response to anti-angiogenic therapy [16]	^ 18^F-FDG (for metabolic activity) ^18^F-FLT (for proliferation) & Cu-ATSM PET (for hypoxia) and CT	Model based on previous work [15] with an added vascular component. Microvessel density was used as model parameter in direct relationship with the vascular growth fraction. Probability density functions were used to sample capillary properties and geometry.	The maximum vascular growth fraction was found to be the most sensitive model parameter. The dosage of the anti-angiogenic agent bevacizumab can be adjusted to improve oxygenation. The model was validated on imaging data of a phase I trial with bevacizumab on head and neck cancer patients.
